# Spatiotemporal Characteristics and Risk Factors for All and Severity-Specific Preterm Births in Southern China, 2014-2021: Large Population-Based Study

**DOI:** 10.2196/48815

**Published:** 2024-06-18

**Authors:** Huazhang Miao, Hui He, Chuan Nie, Jianbing Ren, Xianqiong Luo

**Affiliations:** 1 Department of Healthcare Guangdong Women and Children Hospital Guangzhou China; 2 Department of Pediatric Endocrinology and Inherited Metabolic Diseases Guangdong Women and Children Hospital Guangzhou China; 3 Neonatology Department Guangdong Women and Children Hospital Guangzhou China

**Keywords:** preterm birth, spatiotemporal, incidence, risk, neonatal, infant, pregnancy health, pregnancy complication, pregnancy, birth defect, birth defects, obstetric labor, premature

## Abstract

**Background:**

The worldwide incidence of preterm births is increasing, and the risks of adverse outcomes for preterm infants significantly increase with shorter gestation, resulting in a substantial socioeconomic burden. Limited epidemiological studies have been conducted in China regarding the incidence and spatiotemporal trends of preterm births. Seasonal variations in risk indicate the presence of possible modifiable factors. Gender influences the risk of preterm birth.

**Objective:**

This study aims to assess the incidence rates of preterm birth, very preterm birth, and extremely preterm birth; elucidate their spatiotemporal distribution; and investigate the risk factors associated with preterm birth.

**Methods:**

We obtained data from the Guangdong Provincial Maternal and Child Health Information System, spanning from January 1, 2014, to December 31, 2021, pertaining to neonates with gestational ages ranging from 24 weeks to 42 weeks. The primary outcome measures assessed variations in the rates of different preterm birth subtypes over the course of the study, such as by year, region, and season. Furthermore, we examined the relationship between preterm birth incidence and per capita gross domestic product (GDP), simultaneously analyzing the contributing risk factors.

**Results:**

The analysis incorporated data from 13,256,743 live births. We identified 754,268 preterm infants and 12,502,475 full-term infants. The incidences of preterm birth, very preterm birth, and extremely preterm birth were 5.69 per 100 births, 4.46 per 1000 births, and 4.83 per 10,000 births, respectively. The overall incidence of preterm birth increased from 5.12% in 2014 to 6.38% in 2021. The incidence of extremely preterm birth increased from 4.10 per 10,000 births in 2014 to 8.09 per 10,000 births in 2021. There was a positive correlation between the incidence of preterm infants and GDP per capita. In more developed economic regions, the incidence of preterm births was higher. Furthermore, adjusted odds ratios revealed that advanced maternal age, multiple pregnancies, and male infants were associated with an increased risk of preterm birth, whereas childbirth in the autumn season was associated with a protective effect against preterm birth.

**Conclusions:**

The incidence of preterm birth in southern China exhibited an upward trend, closely linked to enhancements in the care capabilities for high-risk pregnant women and critically ill newborns. With the recent relaxation of China's 3-child policy, coupled with a temporary surge in advanced maternal age and multiple pregnancies, the risk of preterm birth has risen. Consequently, there is a pressing need to augment public health investments aimed at mitigating the risk factors associated with preterm birth, thereby alleviating the socioeconomic burden it imposes.

## Introduction

Neonatal diseases constitute the primary cause of mortality in children younger than 5 years, comprising approximately 37.7% of such deaths [1]. Globally, preterm birth (PTB) stands as the most significant risk factor for neonatal morbidity and mortality [2,3], contributing to around 18% of all fatalities in children younger than 5 years and as much as 35% of all fatalities among neonates (aged <28 days) [4]. The United Nations Sustainable Development Goal (SDG) 3.2 for 2030 aims to eliminate preventable child mortality worldwide, targeting a reduction of neonatal mortality to below 12 per 1000 live births and under-5 mortality to below 25 per 1000 live births. Data indicate that, although the global under-5 mortality rate declined by almost one-half, decreasing from 71.2 per 1000 to 37.1 per 1000 between 2000 and 2019, the neonatal mortality rate exhibited a slower reduction, dropping from 28.0 per 1000 to 17.9 per 1000. Notably, 32% of countries are projected to fall short of attaining any of the SDG 3.2 targets by 2030 [1]. Hence, directing attention to health care systems in regions experiencing a high prevalence of PTBs, augmenting investments in public health and medical interventions to mitigate PTB, and enhancing perinatal and neonatal care in focal areas may contribute to diminishing child mortality, progressively aligning with the 2030 SDGs.

The World Health Organization (WHO) defines PTB as the delivery of newborns before 37 completed weeks of gestation. Over the past 2 decades, there has been a global increase in the incidence of PTB, resulting in a global PTB rate of 11% [5]. In 2022, data from the US National Vital Statistics indicated that the rate of PTB for singletons was 10.38% [6]. Twins constitute 20% of all preterm deliveries, with 60% and 10.7% of twins delivered before 37 and 32 weeks of gestation, respectively [7]. Twin pregnancies are associated with a higher risk of PTB than singleton pregnancies. A meta-analysis conducted in China revealed that the pooled rate of preterm delivery in hospital-based studies was 7.2% (95% CI 6.9%-7.6%), whereas the population-based rates were 5.3% for eastern, 4.6% for central, and 3.8% for western regions [8]. Six countries—India, China, Nigeria, Pakistan, Indonesia, and the United States—collectively contribute to 45.9% (approximately 7.4 million) of the total global PTBs, and it is projected that China will have a PTB rate of around 6.9% [2]. In a study conducted in the United States concerning mortality, in-hospital morbidity, and a 2-year follow-up of extremely preterm infants, 78.3% survived until discharge. Among newborns delivered at gestational ages below 27 weeks, rehospitalization and neurodevelopmental disorders were more prevalent at 2 years of age [9]. This underscores that PTB constitutes a significant health care challenge and a substantial socioeconomic burden [4].

Our study specifically investigated the spatiotemporal aspects and risk factors associated with PTB in 21 cities in southern China from 2014 to 2021. This research aimed to guide the development of public health and health care policies that address regional health equity and balanced development.

## Methods

### Data Sources and Data Collection

The data for this study were sourced from the Maternal and Child Health Information System of Guangdong Province, China. This system comprehensively records data on all births, encompassing information such as place of birth, date of birth, gender, gestational week, weight, length, and maternal age. Hospital staff or midwives input this data into the system based on the babies’ medical records, which are further reviewed by obstetricians. The retrospective data set included information on birth weight, gender, delivery method, maternal age, and household registration of infants born between 24 weeks and 42 weeks of gestation from January 1, 2014, to December 31, 2021, and used fuzzy identity information. The data were reported and registered by medical personnel in 21 cities in southern China, achieving an almost 100% coverage rate of live births. Midwives used calibrated electronic scales to measure birth weight within 30 minutes of delivery, ensuring the recording of stable data.

Estimating gestational age during pregnancy typically entails the integration of both last menstrual period and ultrasound data. When data sources exhibit a significant prevalence of errors in gestational age or when last menstrual period determination is unfeasible, ultrasound measurements are used to precisely ascertain the gestational age [10,11].

In this study, we categorized the 21 cities in southern China into 4 areas based on their geographical location and economic conditions: Pearl River Delta (PRD), eastern Guangdong, western Guangdong, and northern Guangdong. The PRD region comprises 9 cities: Guangzhou, Shenzhen, Foshan, Dongguan, Zhuhai, Zhongshan, Jiangmen, Zhaoqing, and Huizhou. The eastern Guangdong region encompasses 4 cities: Chaozhou, Shantou, Shanwei, and Jieyang. The western Guangdong region consists of 3 cities: Zhanjiang, Maoming, and Yangjiang. The northern Guangdong region comprises 5 cities: Shaoguan, Heyuan, Meizhou, Qingyuan, and Yunfu. Additionally, in this context, spring corresponds to March 1 to May 31, summer corresponds to June 1 to August 31, autumn corresponds to September 1 to November 30, and winter corresponds to December 1 to February 28/29 of the respective year.

### PTB Categorization

PTB refers to newborns delivered before completing 37 weeks of pregnancy, with very preterm birth (VPTB) defined as occurring before 32 completed weeks of gestation and extremely preterm birth (EPTB) defined as before 28 completed weeks of gestation [12].

### Ethical Considerations

This study received approval from the Ethics Committee of Guangdong Women and Children Hospital (approval number: 202201332). The study relied on routine registration data and did not involve patients’ participation in questionnaires, outcome assessments, and other research methods. Additionally, privacy information such as personal identity and contact details have been concealed. The requirement for informed consent was waived by the hospital’s ethics review committee.

### Statistical Analysis

Maternal and neonatal characteristics are presented as a mean (SD) for continuous variables and number (%) for categorical variables. Independent samples Satterthwaite *t* tests were conducted to examine differences in the means of continuous variables. Prevalences of total PTBs (<37 completed weeks of gestation, per 100 births), VPTBs (<32 completed weeks of gestation, per 1000 births), and EPTBs (<28 completed weeks of gestation, per 10,000 births) according to the year, area of residence, region of Guangdong, season of delivery, maternal age, neonatal gender, and number of fetuses were calculated using a binomial distribution exact method. Chi-square tests were used to compare prevalences and obtain the *P* value.

Crude or adjusted associations between PTB and season of delivery, maternal age, neonatal gender, and number of fetuses were examined separately using a logistic regression model. The univariable model included PTB as the dependent variable and included only season of delivery, maternal age, neonatal gender, and number of fetuses as separate independent variables. The multivariable model included PTB as the dependent variable and adjusted for season of delivery, maternal age, neonatal gender, and number of fetuses.

Analyses were done using SAS 9.4 (SAS Institute) and R version 4.2.1 (R Core Team) for the figures. A 2-sided *P*<.05 was considered statistically significant.

## Results

We conducted an analysis of 13,501,312 infants delivered between January 1, 2014, and December 31, 2021, in 21 cities in southern China and spanning gestational ages from 24 weeks to 45 weeks (Multimedia Appendix 1). Exclusions from the data analysis were for the following reasons: 17,794 cases (0.13%) involved non-Chinese pregnant women, 148,351 cases (1.10%) had non-Chinese fathers, 9812 cases (0.07%) were not live births, 56 cases (<0.01%) had an unspecified gender, 6017 cases (0.04%) had a maternal age outside the range of 15 years to 60 years or a missing maternal age, 55,047 (<0.41%) had a paternal age beyond the range of 15 years to 60 years or a missing paternal age, 333 cases (<0.01%) had a birth weight outside the range of 350 g to 6000 g, and 7159 cases (0.05%) had a gestational age outside the range of 16 weeks to 45 weeks. The data set included a total of 13,256,743 live births (Multimedia Appendix 2).

Table 1 provides an overview of the characteristics of the live-born infants included in the analysis. The conception rate varied significantly by season, with winter showing a relatively higher proportion, at approximately 32.68% (4,332,432/13,256,743), while autumn had a higher rate of childbirth, at 27.96% (3,706,674/13,256,743). Over the period spanning from 2014 to 2021, there were 754,268 preterm infants and 12,502,475 full-term infants. Among the PTBs, 419,314 (55.59%) occurred among Aboriginal inhabitants, and a significantly larger proportion occurred in urban areas (89.86%) compared with rural areas. Notably, within the PRD region, there were 472,468 PTBs, constituting a substantially higher percentage (472,468/754,268, 62.64%) than in the eastern, western, and northern regions of Guangdong.

**Table 1:** Comparison of maternal and neonatal characteristics between preterm births (<37 completed weeks of gestation; n=754,268) and term births (≥37 completed weeks of gestation; n=12,502,475) in southern China^a^, 2014-2021 (N=13,256,743).

Characteristics		Preterm births	Term births	Total	Statistical test results (*df*)		*P* value	
**Area of residence, n (%)**						9825.20 (1)^b^		<.001
	Urban	677,772 (89.86)	10,724,873 (85.78)	11,402,645 (86.01)				
	Rural	76,496 (10.14)	1,777,602 (14.22)	1,854,098 (13.99)				
**Household registration, n (%)**						3920.14 (1)^b^		<.001
	Local	419,314 (55.59)	7,406,819 (59.24)	7,826,133 (59.04)				
	Nonlocal	334,954 (44.41)	5,095,656 (40.76)	5,430,610 (40.96)				
**Region of GD^c^, n (%)**						21,462.59 (3)^b^		<.001
	PRD^d^	472,468 (62.64)	6,834,971 (54.67)	7,307,439 (55.12)				
	Eastern GD	85,806 (11.38)	1,982,067 (15.85)	2,067,873 (15.60)				
	Western GD	97,305 (12.90)	1,983,107 (15.86)	2,080,412 (15.69)				
	Northern GD	98,689 (13.08)	1,702,330 (13.62)	1,801,019 (13.59)				
**Season of conception, n (%)**						2225.09 (3)^b^		<.001
	Spring	189,427 (25.11)	2,848,584 (22.78)	3,038,011 (22.92)				
	Summer	155,520 (20.62)	2,631,794 (21.05)	2,787,314 (21.03)				
	Autumn	172,078 (22.81)	2,926,908 (23.41)	3,098,986 (23.38)				
	Winter	237,243 (31.45)	4,095,189 (32.76)	4,332,432 (32.68)				
**Season of delivery, n (%)**						1492.84 (3)^b^		<.001
	Spring	172,829 (22.91)	2,856,963 (22.85)	3,029,792 (22.85)				
	Summer	192,966 (25.58)	3,144,972 (25.15)	3,337,938 (25.18)				
	Autumn	197,651 (26.20)	3,509,023 (28.07)	3,706,674 (27.96)				
	Winter	190,822 (25.30)	2,991,517 (23.93)	3,182,339 (24.01)				
Maternal age (years), mean (SD)		29.18 (5.59)	28.05 (5.03)	28.12 (5.07)	170.44 (829,409)^e^		<.001	
**Maternal age (years** **), n (%)**						29,772.81 (1)^b^		<.001
	<35	622,431 (82.52)	11,128,746 (89.01)	11,751,177 (88.64)				
	≥35	131,837 (17.48)	1,373,729 (10.99)	1,505,566 (11.36)				
Paternal age (years), mean (SD)		31.56 (6.14)	30.43 (5.58)	30.49 (5.62)	155.98 (831,145)^e^		<.001	
**Paternal age (years), n (%)**						24,363.23 (1)^b^		<.001
	<35	538,114 (71.34)	9,869,950 (78.94)	10,408,064 (78.51)				
	≥35	216,154 (28.66)	2,632,525 (21.06)	2,848,679 (21.49)				
**Method of delivery, n (%)**						112,969.00 (2)^b^		<.001
	Vaginal	348,215 (46.17)	7,822,312 (62.57)	8,170,527 (61.63)				
	Cesarean section	321,930 (42.68)	3,166,736 (25.33)	3,488,666 (26.32)				
	Unknown	84,123 (11.15)	1,513,427 (12.11)	1,597,550 (12.05)				
**Neonatal gender, n (%)**						6541.60 (1)^b^		<.001
	Male	436,768 (57.91)	6,641,625 (53.12)	7,078,393 (53.39)				
	Female	317,500 (42.09)	5,860,850 (46.88)	6,178,350 (46.61)				
**Number of previous childbirths, n (%)**						1941.49 (2)^b^		<.001
	1	373,655 (49.54)	6,410,764 (51.28)	6,784,419 (51.18)				
	2	271,527 (36.00)	4,491,143 (35.92)	4,762,670 (35.93)				
	≥3	109,086 (14.46)	1,600,568 (12.80)	1,709,654 (12.90)				
**Number of fetuses, n (%)**						1,133,440.00 (2)^b^		<.001
	1	599,814 (79.52)	12,342,371 (98.72)	12,942,18 5 (97.63)				
	2	152,342 (20.20)	159,039 (1.27)	311,381 (2.35)				
	≥3	2112 (0.28)	1065 (0.01)	3117 (0.02)				
Gestational age (weeks), mean (SD)		34.66 (1.92)	39.00 (1.10)	38.76 (1.53)	1946.70 (784,261)^e^		<.001	
**Gestational age (weeks), n (%)**						13,256,743.00 (4)^b^		<.001
	<28	6399 (0.85)	0 (0.00)	6399 (0.05)				
	28-32	84,797 (11.24)	0 (0.00)	84,797 (0.64)				
	33-36	663,072 (87.91)	0 (0.00)	663,072 (5.00)				
	37-41	0 (0.00)	12,424,849 (99.38)	12,424,849 (93.72)				
	≥42	0 (0.00)	77,626 (0.62)	77,626 (0.59)				
Birth weight (g), mean (SD)		2381.40 (519.20)	3200.40 (398.10)	3153.76 (448.06)	1346.10 (808,645)^e^		<.001	
**Birth weight (g), n (%)**						3,808,626.00 (4)^b^		<.001
	<1000	6170 (0.82)	108 (0.00)	6278 (0.05)				
	1000-1499	38,543 (5.11)	972 (0.01)	39,515 (0.30)				
	1500-2499	362,945 (48.12)	316,810 (2.53)	679,755 (5.13)				
	2500-3999	345,227 (45.77)	11,775,742 (94.19)	12,120,969 (91.43)				
	≥4000	1383 (0.18)	408,843 (3.27)	410,226 (3.09)				

^a^Four regions (Pearl River Delta region and eastern, western, and northern Guangdong regions) were included.

^b^Chi-square test.

^c^GD: Guangdong.

^d^PRD: Pearl River Delta.

^e^Satterthwaite *t* test.

Additionally, Table 1 presents data on the average maternal age, which was 28.12 (SD 5.07) years, with an 11.36% (1,505,566/13,256,743) rate of advanced maternal age (≥35 years old), and the average paternal age, which was 30.49 (SD 5.62) years, with a 21.49% (2,848,679/13,256,743) rate of advanced paternal age (≥35 years old). In their first birth, 373,655 women delivered a preterm baby. Among PTB, 79.52% (599,814/754,268) were singleton pregnancies. The average gestational age of infants was 38.76 (SD 1.53) weeks, with an average birth weight of 3153.76 (SD 448.06) g. In contrast, preterm infants had an average gestational age of 34.66 (SD 1.92) weeks and an average birth weight of 2381.40 (SD 519.20) g.

**Figure 1:**
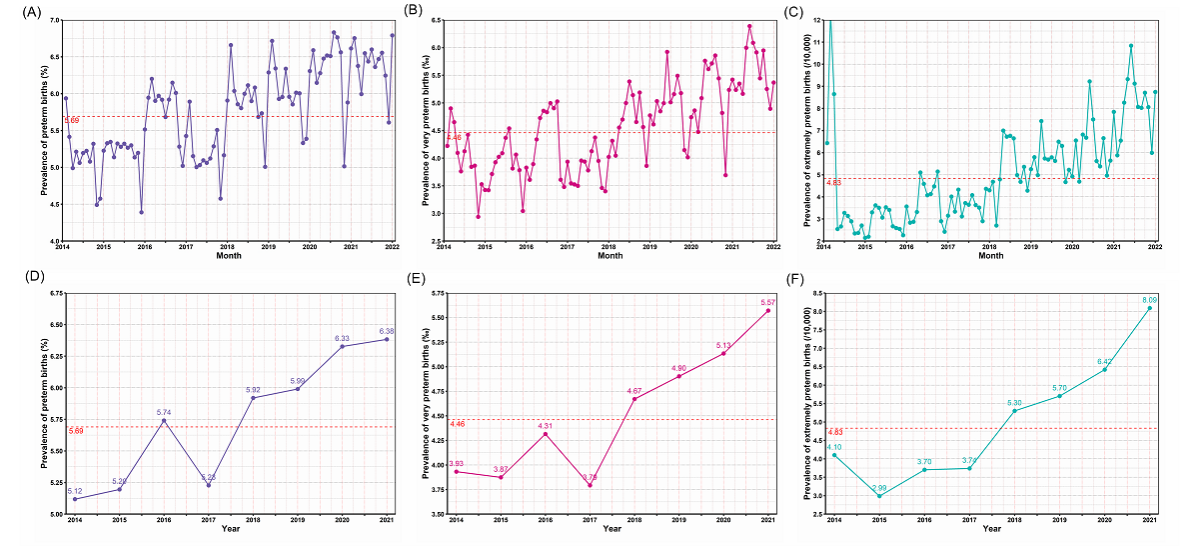
In southern China, 2014-2021: (A) monthly prevalence of preterm births (PTBs; <37 completed weeks of gestation, per 100 births); (B) monthly prevalence of very preterm births (VPTBs; <32 completed weeks of gestation, per 1000 births); (C) monthly prevalence of extremely preterm births (EPTBs; <28 completed weeks of gestation, per 10,000 births); (D) yearly prevalence of PTBs (<37 completed weeks of gestation, per 100 births); (E) yearly prevalence of VPTBs (<32 completed weeks of gestation, per 1000 births); (F) yearly prevalence of EPTBs (<28 completed weeks of gestation, per 10,000 births).

Figure 1 illustrates the temporal trend in the 3 distinct categories of preterm infants from 2014 to 2021. PTB, VPTB, and EPTB increased year on year. PTB increased from 5.12 (per 100 births) in 2014 to 6.38 (per 100 births) in 2021, VPTB rose from 3.93 (per 1000 births) in 2014 to 4.46 (per 1000 births) in 2021, and EPTB surged from 4.10 (per 10,000 births) in 2014 to 8.09 (per 10,000 births) in 2021. Notably, a marked reduction in the incidence of preterm infants was evident at the start of the subsequent year, with the rates of PTB and VPTB experiencing a sharp increase in 2016 followed by a decline in 2017.

**Figure 2:**
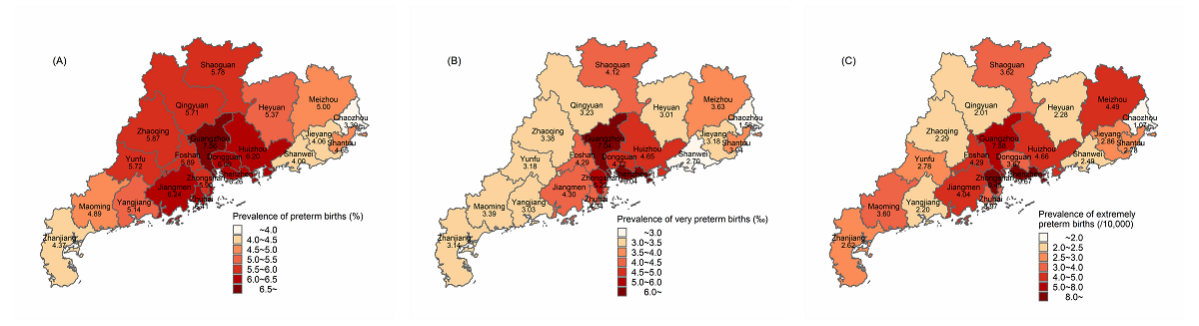
In southern China, 2014-2021: (A) spatial distributions of the prevalence of preterm births (<37 completed weeks of gestation, per 100 births); (B) spatial distributions of the prevalence of very preterm births (<32 completed weeks of gestation, per 1000 births); (C) spatial distributions of the prevalence of extremely preterm births (<28 completed weeks of gestation, per 10,000 births).

Figure 2 and Multimedia Appendix 3 spatially delineate the 3 distinct categories of preterm infants. Generally, PTB rates across all types are higher in the PRD region, with Guangzhou, as the provincial capital, having the highest prevalence. It is noteworthy that, in the case of EPTB, the cities with higher quantities of PTBs are not predominantly concentrated in the PRD region. Additionally, there are 1 to 2 cities in eastern, western, and northern Guangdong with high EPTB rates.

**Figure 3:**
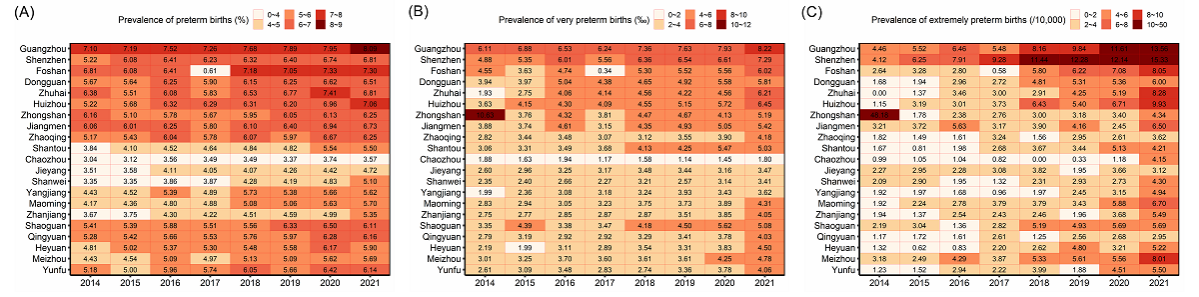
Across 21 cities in southern China, 2014-2021: heat maps of the yearly prevalences of (A) preterm births (<37 completed weeks of gestation, per 100 births); (B) very preterm births (<32 completed weeks of gestation, per 1000 births); (C) extremely preterm births (<28 completed weeks of gestation, per 10,000 births).

Figure 3 provides a detailed description of the incidence and temporal trends of preterm infants in 21 cities within southern China. Irrespective of the type of PTB, the rate was lower in eastern Guangdong than in the other regions. For both VPTB and EPTB, the rates in Guangzhou and Shenzhen were notably high, with the incidence of EPTB in Shenzhen continuously increasing relative to Guangzhou since 2015.

Table 2 provides a comparative analysis of the incidences of various types of preterm infants. Over the period of 2014 to 2021, the incidence rates for PTB, VPTB, and EPTB were 5.69 (per 100 births), 4.46 (per 1000 births), and 4.83 (per 10,000 births), respectively. All 3 types of preterm infants occurred more frequently in urban areas than in rural regions. It is noteworthy that the 3 types of preterm infants have the lowest birth rates in the autumn season. Additionally, advanced maternal age (≥35 years) and multiple pregnancies posed a higher risk for PTB, regardless of PTB, VPTB, or EPTB. Furthermore, PTB is significantly more common in boys than in girls.

**Table 2:** Prevalence of total preterm births (<37 completed weeks of gestation, per 100 births), very preterm births (<32 completed weeks of gestation, per 1000 births), and extremely preterm births (<28 completed weeks of gestation, per 10,000 births) in southern China, 2014-2021.

Subgroups		Births, n	Total preterm births					Very preterm births						Extremely preterm births			
			Results, n	Prevalence (95% CI)	*P* value^a^		Results, n		Prevalence (95% CI)	*P* value^a^		Results, n			Prevalence (95% CI)	*P* value^a^	
**Years**						<.001					<.001						<.001
	2014	1,816,688	92,985	5.12 (5.09-5.15)			7143		3.93 (3.84-4.02)			745			4.10 (3.81-4.41)		
	2015	1,662,742	86,386	5.20 (5.16-5.23)			6441		3.87 (3.78-3.97)			497			2.99 (2.73-3.26)		
	2016	1,876,740	107,738	5.74 (5.71-5.77)			8098		4.31 (4.22-4.41)			695			3.70 (3.43-3.99)		
	2017	1,906,491	99,665	5.23 (5.20-5.26)			7230		3.79 (3.71-3.88)			713			3.74 (3.47-4.02)		
	2018	1,699,744	100,617	5.92 (5.88-5.96)			7939		4.67 (4.57-4.77)			901			5.30 (4.96-5.66)		
	2019	1,637,556	98,089	5.99 (5.95-6.03)			8028		4.90 (4.80-5.01)			934			5.70 (5.34-6.08)		
	2020	1,409,671	89,182	6.33 (6.29-6.37)			7238		5.13 (5.02-5.25)			905			6.42 (6.01-6.85)		
	2021	1,247,111	79,606	6.38 (6.34-6.43)			6948		5.57 (5.44-5.70)			1009			8.09 (7.6-8.61)		
	2014-2021	13,256,743	754,268	5.69 (5.68-5.70)			59,065		4.46 (4.42-4.49)			6399			4.83 (4.71-4.95)		
**Area of residence**						<.001					<.001						<.001
	Urban	11,402,645	677,772	5.94 (5.93-5.96)			55,539		4.87 (4.83-4.91)			6112			5.36 (5.23-5.50)		
	Rural	1,854,098	76,496	4.13 (4.10-4.15)			3526		1.90 (1.84-1.97)			287			1.55 (1.37-1.74)		
**Region of GD^b^**						<.001					<.001						<.001
	PRD^c^	7,307,439	472,468	6.47 (6.45-6.48)			39,706		5.43 (5.38-5.49)			4705			6.44 (6.26-6.63)		
	Eastern GD	2,067,873	85,806	4.15 (4.12-4.18)			6476		3.13 (3.06-3.21)			527			2.55 (2.34-2.78)		
	Western GD	2,080,412	97,305	4.68 (4.65-4.71)			6702		3.22 (3.14-3.30)			612			2.94 (2.71-3.18)		
	Northern GD	1,801,019	98,689	5.48 (5.45-5.51)			6181		3.43 (3.35-3.52)			555			3.08 (2.83-3.35)		
**Season of delivery**						<.001					<.001						<.001
	Spring^d^	3,029,792	172,829	5.70 (5.68-5.73)			14,163		4.67 (4.60-4.75)			1668			5.51 (5.24-5.78)		
	Summer^e^	3,337,938	192,966	5.78 (5.76-5.81)			16,090		4.82 (4.75-4.90)			1644			4.93 (4.69-5.17)		
	Autumn^f^	3,706,674	197,651	5.33 (5.31-5.36)			15,208		4.10 (4.04-4.17)			1559			4.21 (4.00-4.42)		
	Winter^g^	3,182,339	190,822	6.00 (5.97-6.02)			13,604		4.27 (4.20-4.35)			1528			4.80 (4.56-5.05)		
**Maternal age (years)**						<.001					<.001						<.001
	<35	11,751,177	622,431	5.30 (5.28-5.31)			47,353		4.03 (3.99-4.07)			4891			4.16 (4.05-4.28)		
	≥35	1,505,566	131,837	8.76 (8.71-8.80)			11,712		7.78 (7.64-7.92)			1508			10.02 (9.52-10.53)		
**Neonatal gender**						<.001					<.001						<.001
	Male	7,078,393	436,768	6.17 (6.15-6.19)			34,985		4.94 (4.89-4.99)			3959			5.59 (5.42-5.77)		
	Female	6,178,350	317,500	5.14 (5.12-5.16)			24,080		3.90 (3.85-3.95)			2440			3.95 (3.79-4.11)		
**Number of fetuses**						<.001					<.001						<.001
	1	12,942,185	599,814	4.63 (4.62-4.65)			45,393		3.51 (3.48-3.54)			4611			3.56 (3.46-3.67)		
	2	311,381	152,342	48.92 (48.75-49.10)			13,228		42.48 (41.78-43.20)			1723			55.33 (52.76-58.00)		
	≥3	3177	2112	66.48 (64.81-68.12)			444		139.75 (127.88-152.30)			65			204.6 (158.25-260.04)		

^a^Chi-square tests were used to compare the prevalences for all of these subgroups.

^b^GD: Guangdong.

^c^PRD: Pearl River Delta.

^d^Includes March, April, and May.

^e^Includes June, July, and August.

^f^Includes September, October, and November.

^g^Includes December, January, and February.

**Figure 4:**
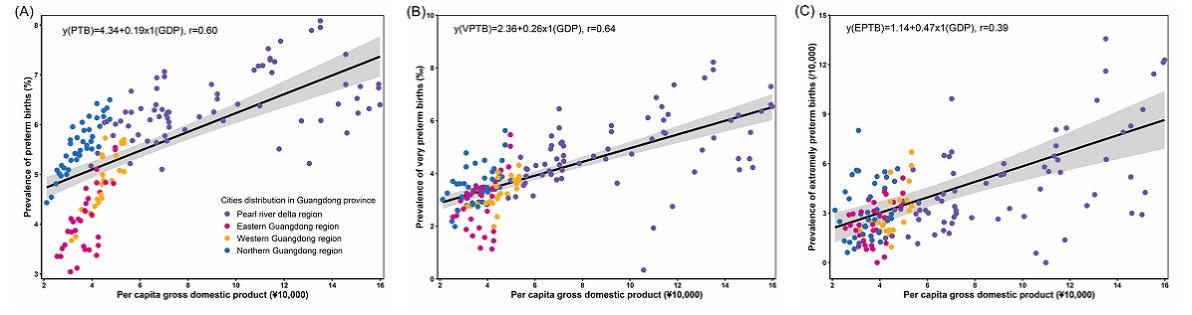
Across 21 cities in southern China, 2014-2021: correlations between per capita gross domestic product (GDP) and (A) preterm births (PTB; <37 completed weeks of gestation, per 100 births); (B) very preterm births (VTPB; <32 completed weeks of gestation, per 1000 births); (C) extremely preterm births (EPTB; <28 completed weeks of gestation, per 10,000 births).

Figure 4 illustrates the association between the incidence of the 3 distinct types of preterm infants and per capita gross domestic product (GDP). As the economy developed, there was an increase in the incidences of PTB, VPTB, and EPTB. Notably, the positive correlation between VPTB and GDP per capita was the most pronounced (*r*=0.64).

Table 3 examines the risk factors associated with the 3 categories of preterm infants. The adjusted odds ratios (ORs) revealed that advanced maternal age (adjusted OR 1.59, 95% CI 1.58-1.60), multiple pregnancies (adjusted OR 40.52, 95% CI 37.63-43.64), and male infants (adjusted OR 1.24, 95% CI 1.23-1.25) were risk factors for PTB. In contrast, giving birth in the autumn season was found to be a protective factor against PTB.

**Table 3:** Risk factors for total preterm births (<37 completed weeks of gestation), very preterm births (<32 completed weeks of gestation), and extremely preterm births (<28 completed weeks of gestation) in southern China, 2014-2021.

Subgroup			Preterm births									Very preterm births									Extremely preterm births						
			Crude OR^a^ (95% CI)		*P* value		Adjusted^b^ OR (95% CI)		*P* value		Crude OR (95% CI)			*P* value		Adjusted^b^ OR (95% CI)		*P* value		Crude OR (95% CI)			*P* value		Adjusted^b^ OR (95% CI)		*P* value
**Season of delivery**																											
	Spring	1.07 (1.07-1.08)		<.001		1.04 (1.03-1.05)		<.001		1.14 (1.11-1.17)			<.001		1.10 (1.07-1.13)		<.001		1.31 (1.22-1.40)			<.001		1.25 (1.17-1.34)		<.001	
	Summer	1.09 (1.08-1.10)		<.001		1.06 (1.05-1.07)		<.001		1.18 (1.15-1.20)			<.001		1.14 (1.11-1.16)		<.001		1.17 (1.09-1.26)			<.001		1.12 (1.05-1.20)		<.001	
	Autumn	Reference		—^c^		Reference		—		Reference			—		Reference		—		Reference			—		Reference		—	
	Winter	1.13 (1.13-1.14)		<.001		1.11 (1.10-1.12)		<.001		1.04 (1.02-1.07)			<.001		1.01 (0.99-1.04)		.362		1.14 (1.06-1.23)			.362		1.10 (1.02-1.18)		<.001	
**Maternal age (years)**																											
	<35	Reference		—		Reference		—		Reference			—		Reference		—		Reference			—		Reference		—	
	≥35	1.72 (1.71-1.73)		<.001		1.59 (1.58-1.60)		<.001		1.94 (1.90-1.98)			<.001		1.70 (1.67-1.74)		<.001		2.41 (2.27-2.55)			<.001		2.04 (1.93-2.16)		<.001	
**Neonatal gender**																											
	Male	1.21 (1.21-1.22)		<.001		1.24 (1.23-1.25)		<.001		1.27 (1.25-1.29)			<.001		1.27 (1.25-1.29)		<.001		1.42 (1.35-1.49)			<.001		1.41 (1.35-1.49)		<.001	
	Female	Reference		—		Reference		—		Reference			—		Reference		—		Reference			—		Reference		—	
**Number of fetuses**																											
	1	Reference		—		Reference		—		Reference			—		Reference		—		Reference			—		Reference		—	
	2	19.71 (19.56-18.86)		<.001		19.39 (19.25-19.54)		<.001		12.61 (12.36-12.86)			<.001		12.06 (11.82-12.30)		<.001		15.62 (14.78-16.51)			<.001		14.60 (13.80-15.44)		<.001	
	≥3	40.61 (37.72-43.71)		<.001		40.52 (37.63-43.64)		<.001		46.16 (41.74-51.05)			<.001		44.61 (40.32-49.35)		<.001		58.62 (45.78-75.07)			<.001		55.38 (43.23-70.95)		<.001	

^a^OR: odds ratio.

^b^Multivariate adjustment for season of delivery, maternal age, neonatal sex, and number of fetuses.

^c^Not applicable.

## Discussion

### Principal Findings

As medical technology continues to advance, the Chinese 4-level maternal and child health service network and 2-way referral systems have played significant roles in the gradual increase of the survival rate among PTBs. In this study, we analyzed and reviewed the epidemiological trends and risk factors associated with preterm infants in 21 cities in southern China over the past 8 years, using the largest, most recent, and most comprehensive data set available for southern China. These cities, which are densely populated with a significant migrant population, offer a representative sample for assessing premature birth incidence in China. Additionally, our research presents a comprehensive classification of premature infants. The findings indicated a higher rate of PTB in the PRD region, which is characterized by a more developed economy. This not only highlights regional disparities in health care but also points toward directions for enhancing the equity of maternal and child health services and proposing prepregnancy health care guidance. These efforts aim to promote a healthy development model for women and children in the new era in China.

In general, the PTB rate shows a positive correlation with GDP levels, a relationship deeply intertwined with the specific conditions and the maternal and child health system in China. Roughly 15 million preterm infants, representing an adverse pregnancy outcome, are born globally each year. It is worth noting that the rate is as low as 5% even among healthy pregnant women at low risk during pregnancy [13]. Hence, the vast majority of PTBs are linked to multiple pregnancies, pregnancy complications, placental abnormalities, and other contributing factors [14]. Our results support that advanced maternal age and multiple pregnancies are risk factors for the incidence of PTB. Using the maternal and child health service network and referral system in China, the majority of high-risk pregnant women are referred to tertiary hospitals. This results in a scenario in which a significant proportion of preterm infants are born in urban or economically developed regions. Additionally, it is evident that the population of permanent residents in urban areas or more economically developed areas is substantially higher than in less economically developed areas, contributing to the elevated PTB rates in these regions. An investigation spanning from 1990 to 2019 that focused on the global, regional, and national incidences of and mortality associated with neonatal PTB revealed a noteworthy positive correlation between the prevalence of neonatal PTB and the sociodemographic index in 2019, as well as the universal health coverage index in 2019. This correlation was particularly pronounced in high-income countries, such as Greece, Bahrain, Japan, the United Kingdom, and the United States, which corroborates our findings [15].

In October 2015, the Chinese government initiated the universal 2-child policy, which officially took effect in July 2016. The alteration in fertility policy had the potential to result in shifts in the sociodemographic characteristics of pregnant women, including a transient increase in advanced maternal age (≥35 years) [16]. Consequently, the rates of PTBs in 2016 and 2017 notably increased compared with previous years, particularly in the case of EPTB. Despite intensified public health and medical intervention efforts aimed at reducing PTB, the rate has increased in the majority of countries. For instance, PTB rates in the United States increased from 9.98% in 2010 to 10.09% in 2020 [17], while the rate of EPTB decreased from 0.71% to 0.64%. Since 1990, worldwide neonatal mortality has decreased by 37%, and the reduction in the incidence of EPTB has played a role in further lowering neonatal mortality rates [18]. According to survey data from the National Maternal and Child Health Center in China, the incidence of preterm infants in China increased from 5.9% in 2012 to 6.4% in 2018, with an overall rate of approximately 6.1% [16]. The PTB rate in South China also exhibited an upward trend, with the rate of EPTB significantly lower than that in the United States.

The province housing 21 cities in southern China ranks as China’s most densely populated region, boasting a preterm rate significantly below the global average. This achievement can be attributed to the establishment of a robust maternal and child health system and the successful implementation of the national health care reform plan. Nonetheless, there exists a glaring issue of regional health care inequality, exemplified by the concentration of PTBs in the PRD region. Apart from the PRD region, 1 to 2 prominent cities in other regions also had high PTB rates. This suggests that the standard of care for newborns facing severe complication is consistently improving in these leading cities, while health care in other cities remains underdeveloped. Hence, regional management, augmented investment in public health initiatives within these leading cities, and the gradual establishment of a core medical hub centered around these cities represent an appropriate strategy. Ultimately, this approach aims to catalyze the advancement of health care services in the surrounding cities, aims to alleviate the health care burden on provincial capital cities and the PRD areas, and holds significant implications for the promotion of child health in China.

In our study, we observed that season had a notable impact on PTBs. This observation revealed that, although the number of births was higher during autumn than in other seasons, the incidence of PTBs was at its lowest during that time of the year. Simultaneously, our analysis of risk factors suggested that giving birth in autumn (September to November) was associated with a protective effect against PTB. In general, there were 2 peaks in the incidence of PTBs during summer (June 1 to August 31) and winter (December 1 to February 30). These findings align with a Norwegian survey that explored the seasonal impact on PTBs by analyzing data from 2,321,652 birth registrations. When analyzing the underlying reasons for this pattern, it is possible that an increase in unplanned pregnancies during longer summer and winter holidays is a contributing factor. Moreover, individuals with unplanned pregnancies may be less likely to engage in preconception preparation [19].

Specifically, the incidence of PTBs exhibited substantial annual fluctuations, with a notable decrease near the onset of the subsequent year. This phenomenon may be attributed to the subtropical monsoon climate characteristic of southern China, which implies that December has relatively mild temperatures, and such variations in temperature may influence the occurrence of PTBs. Liang et al [20] conducted an analysis of the impact of the 2008 cold spell on PTBs in 2 cities in South China. Regarding the total number of vaginal PTBs, their study revealed that there was a 22.44% increase in Dongguan and a 21.25% increase in Shenzhen during the 2008 cold spell, thereby supporting the influence of a cold spell on PTB. Additionally, a global analysis encompassing 14 lower- to middle-income countries investigated the connection between extreme heat and the occurrence of PTB and stillbirth. The results indicated that higher maximum temperatures and a narrower diurnal temperature range experienced during the last week before birth were associated with an increased risk of PTB and stillbirth [21]. This finding reinforces our findings.

Furthermore, data from 13 studies conducted in the United States and Europe revealed that 54.6% of PTBs were male infants, with a risk ratio of 1.14 (95% CI 1.11-1.17) for PTBs of male infants compared with female infants, aligning with our findings [22]. If the risk ratio for PTB between genders remains consistent within each region and is applied to sex-specific live birth data to estimate sex-specific PTB prevalence, it can offer guidance for future public health investments.

In this study, we investigated the prevalence and risk factors associated with preterm infants in southern China, approaching the analysis both from a spatiotemporal perspective and using a substantial data set. Our findings suggest that, despite a year-on-year increase in the incidence of PTBs, it remains below the global average, which bodes well for the advancement of public health initiatives in China. However, this study has certain limitations. Although the sample size was large, we lacked information on maternal pregnancy complications and general health status, preventing a more in-depth analysis of risk factors and the elimination of potential confounding variables. Additionally, the causal mechanism behind the seasonal influence on PTBs, potentially associated with temperature variations, remains unexplored. In the future, further investigations involving extensive sampling and questionnaire surveys are essential to delve deeply into the impact of climatic factors on PTBs, ultimately contributing to the development of theories for prepregnancy health care.

### Conclusions

The incidence of PTB in South China exhibited an upward trend, closely linked to enhancements in the care capabilities for high-risk pregnant women and critically ill newborns. With the recent relaxation of China’s 3-child policy, coupled with a temporary surge in advanced maternal age and multiple pregnancies, the risk of PTB has risen. Consequently, there is a pressing need to augment public health investments aimed at mitigating the risk factors associated with PTB, thereby alleviating the socioeconomic burden it imposes.

## Appendix

Multimedia Appendix 1 Geographical locations of the study area. Guangdong province is located in South China. This study included all 21 cities in Guangdong province, which are Guangzhou, Shenzhen, Foshan, Dongguan, Zhuhai, Zhongshan, Zhaoqing, Huizhou, Jiangmen, Chaozhou, Shantou, Jieyang, Meizhou, Shanwei, Shaoguan, Heyuan, Qingyuan, Yunfu, Yangjiang, Maoming, and Zhanjiang.

Multimedia Appendix 2 Study flowchart for selection of newborns from birth records in South China, 2014-2021.

Multimedia Appendix 3 Disease distribution maps at sub-county levels: (A) spatial distributions of the prevalence of preterm births (PTB; <37 completed weeks of gestation, per 100 births) in southern China, 2014–2021; (B) spatial distributions of the prevalence of very preterm births (VPTB; <32 completed weeks of gestation, per 1000 births) in southern China, 2014–2021; (C) spatial distributions of the prevalence of extremely preterm births (EPTB; <28 completed weeks of gestation, per 10,000 births) in southern China, 2014-2021.

## Abbreviations

EPTB: extremely preterm birth
GDP: gross domestic product
OR: odds ratio
PRD: Pearl River Delta
PTB: preterm birth
SDG: Sustainable Development Goal
VPTB: very preterm birth
WHO: World Health Organization

## Data Availability

All data generated or analyzed during this study are included in this published article and its supplementary information.

## References

[1] GBD 2019 Under-5 Mortality Collaborators (2021). Global, regional, and national progress towards Sustainable Development Goal 3.2 for neonatal and child health: all-cause and cause-specific mortality findings from the Global Burden of Disease Study 2019. Lancet.

[2] Chawanpaiboon S, Vogel JP, Moller A, Lumbiganon P, Petzold M, Hogan D, Landoulsi S, Jampathong N, Kongwattanakul K, Laopaiboon M, Lewis C, Rattanakanokchai S, Teng DN, Thinkhamrop J, Watananirun K, Zhang J, Zhou W, Gülmezoglu AM (2019). Global, regional, and national estimates of levels of preterm birth in 2014: a systematic review and modelling analysis. Lancet Glob Health.

[3] Goldenberg RL, Culhane JF, Iams JD, Romero R (2008). Epidemiology and causes of preterm birth. Lancet.

[4] Walani SR (2020). Global burden of preterm birth. Int J Gynaecol Obstet.

[5] da Fonseca EB, Damião R, Moreira DA (2020). Preterm birth prevention. Best Pract Res Clin Obstet Gynaecol.

[6] Martin JA, Hamilton BE, Osterman MJK (2023). Births in the United States, 2022. NCHS Data Brief.

[7] Roman A, Ramirez A, Fox NS (2022). Prevention of preterm birth in twin pregnancies. Am J Obstet Gynecol MFM.

[8] Song Q, Chen J, Zhou Y, Li Z, Li H, Liu J (2022). Preterm delivery rate in China: a systematic review and meta-analysis. BMC Pregnancy Childbirth.

[9] Bell EF, Hintz SR, Hansen NI, Bann CM, Wyckoff MH, DeMauro SB, Walsh MC, Vohr BR, Stoll BJ, Carlo WA, Van Meurs KP, Rysavy MA, Patel RM, Merhar SL, Sánchez PJ, Laptook AR, Hibbs AM, Cotten CM, D'Angio CT, Winter S, Fuller J, Das A, Eunice Kennedy Shriver National Institute of Child Health and Human Development Neonatal Research Network (2022). Mortality, in-hospital morbidity, care practices, and 2-year outcomes for extremely preterm infants in the US, 2013-2018. JAMA.

[10] No authors listed (2014). Committee opinion no 611: method for estimating due date. Obstet Gynecol.

[11] Butt K, Lim KI (2019). Guideline No. 388-Determination of gestational age by ultrasound. J Obstet Gynaecol Can.

[12] Gleason CA, Juul SE (2017). Avery's Diseases of the Newborn.

[13] Papageorghiou AT, Ohuma EO, Altman DG, Todros T, Cheikh Ismail L, Lambert A, Jaffer YA, Bertino E, Gravett MG, Purwar M, Noble JA, Pang R, Victora CG, Barros FC, Carvalho M, Salomon LJ, Bhutta ZA, Kennedy SH, Villar J, International Fetal and Newborn Growth Consortium for the 21st Century (INTERGROWTH-21st) (2014). International standards for fetal growth based on serial ultrasound measurements: the Fetal Growth Longitudinal Study of the INTERGROWTH-21st Project. Lancet.

[14] Delnord M, Zeitlin J (2019). Epidemiology of late preterm and early term births - An international perspective. Semin Fetal Neonatal Med.

[15] Cao G, Liu J, Liu M (2022). Global, regional, and national incidence and mortality of neonatal preterm birth, 1990-2019. JAMA Pediatr.

[16] Deng K, Liang J, Mu Y, Liu Z, Wang Y, Li M, Li X, Dai L, Li Q, Chen P, Xie Y, Zhu J, Liu H (2021). Preterm births in China between 2012 and 2018: an observational study of more than 9 million women. Lancet Glob Health.

[17] Osterman M, Hamilton B, Martin JA, Driscoll AK, Valenzuela CP (2021). Births: final data for 2020. Natl Vital Stat Rep.

[18] Lawn JE, Blencowe H, Oza S, You D, Lee ACC, Waiswa P, Lalli M, Bhutta Z, Barros AJD, Christian P, Mathers C, Cousens SN, Lancet Every Newborn Study Group (2014). Every Newborn: progress, priorities, and potential beyond survival. Lancet.

[19] Weinberg CR, Shi M, DeRoo LA, Basso O, Skjærven R (2015). Season and preterm birth in Norway: A cautionary tale. Int J Epidemiol.

[20] Liang Z, Wang P, Zhao Q, Wang B, Ma Y, Lin H, Xiao J, Zhou J (2018). Effect of the 2008 cold spell on preterm births in two subtropical cities of Guangdong Province, Southern China. Sci Total Environ.

[21] McElroy S, Ilango S, Dimitrova A, Gershunov A, Benmarhnia T (2022). Extreme heat, preterm birth, and stillbirth: A global analysis across 14 lower-middle income countries. Environ Int.

[22] Blencowe H, Lee ACC, Cousens S, Bahalim A, Narwal R, Zhong N, Chou D, Say L, Modi N, Katz J, Vos T, Marlow N, Lawn JE (2013). Preterm birth-associated neurodevelopmental impairment estimates at regional and global levels for 2010. Pediatr Res.

