# Supplementary figures and images for: Spatiotemporal Characteristics and Risk Factors for All and Severity-Specific Preterm Births in Southern China, 2014-2021: Large Population-Based Study
Source: JMIR Public Health Surveill. 2024 Jun 18;10:e48815. doi: 10.2196/48815 (PMC11220432; doi:10.2196/48815)

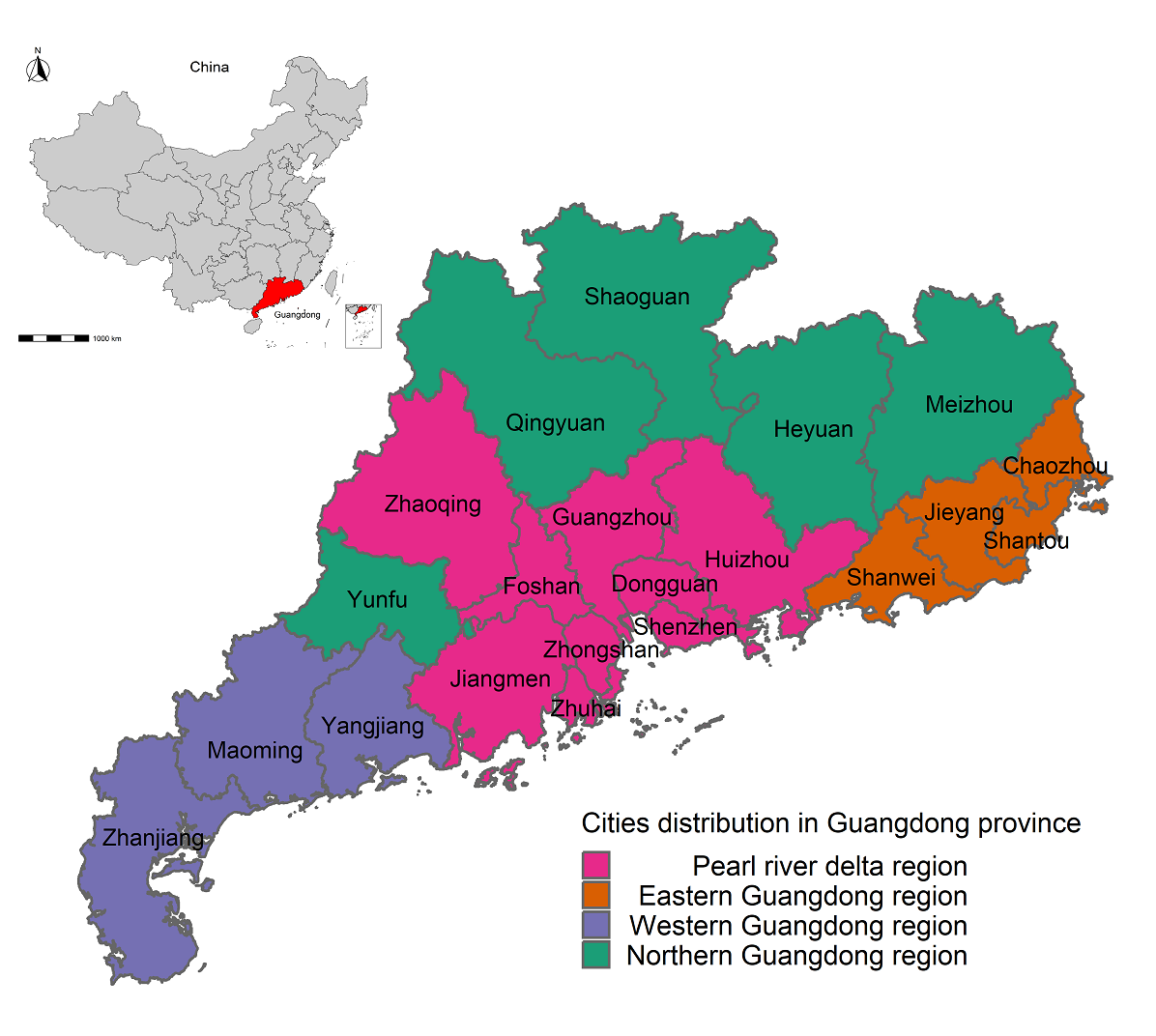

Supplement: Multimedia Appendix 1 [file publichealth_v10i1e48815_app1.png]

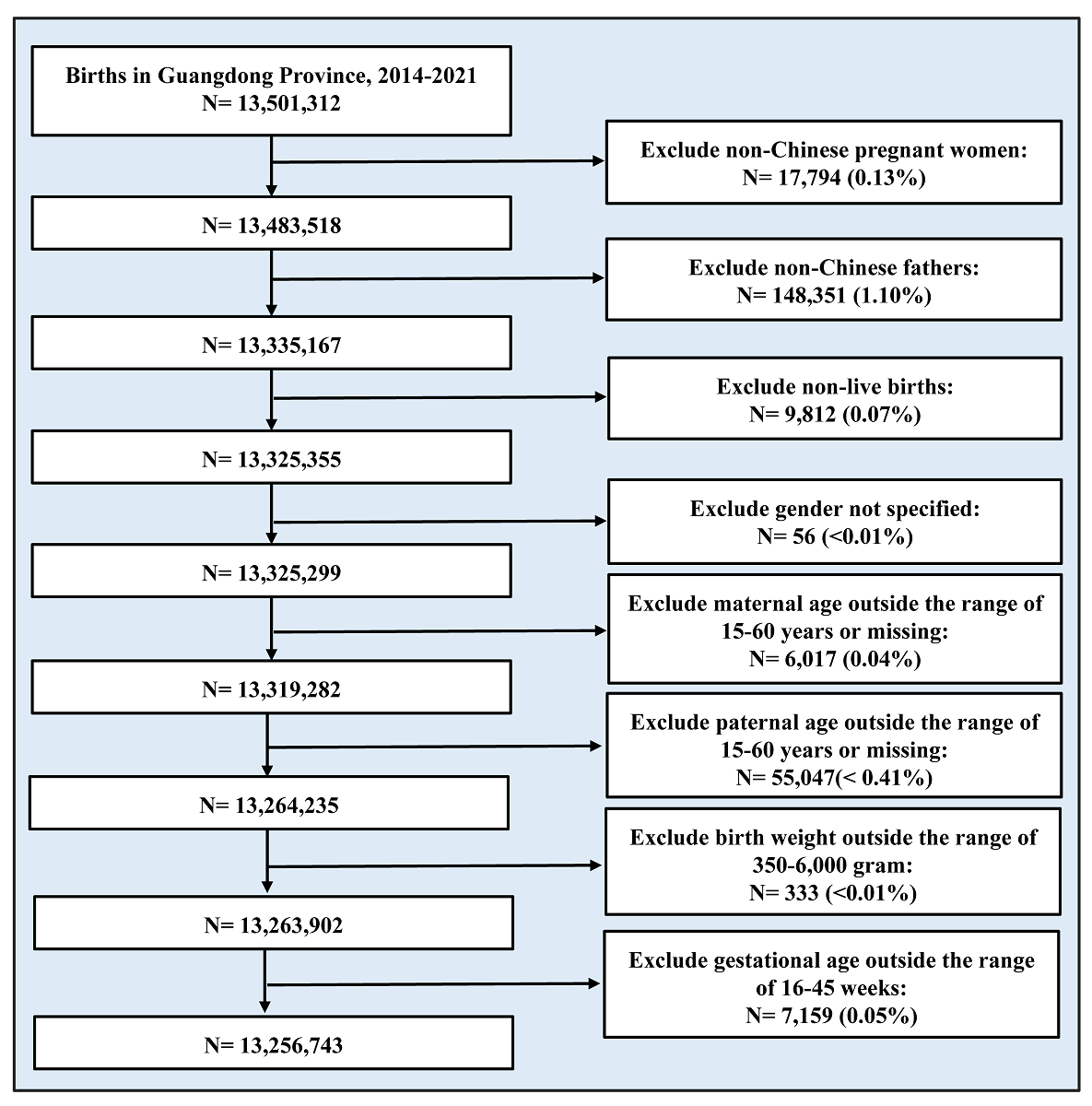

Supplement: Multimedia Appendix 2 [file publichealth_v10i1e48815_app2.png]

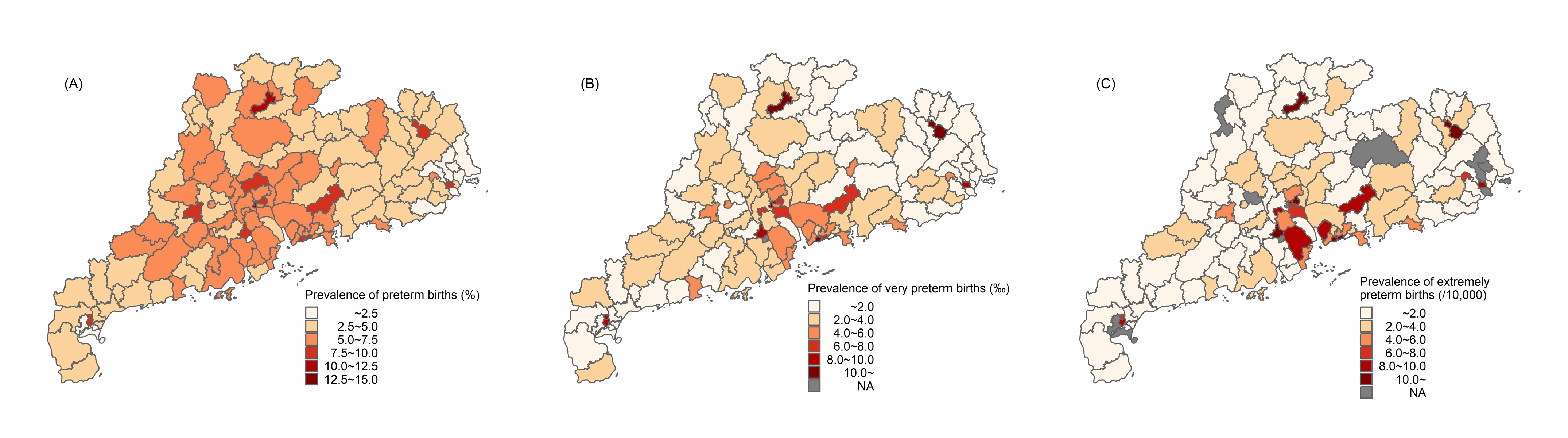

Supplement: Multimedia Appendix 3 [file publichealth_v10i1e48815_app3.png]
